# Plasma Rich in Growth Factors in the Treatment of Endodontic Periapical Lesions in Adult Patients: A Narrative Review

**DOI:** 10.3390/ph14101041

**Published:** 2021-10-13

**Authors:** Agata Zoltowska, Katarzyna Machut, Elzbieta Pawlowska, Marcin Derwich

**Affiliations:** 1 Department of Endodontic Dentistry, Faculty of Medicine, Medical University of Gdansk, 80-210 Gdansk, Poland; azolt@gumed.edu.pl (A.Z.); katarzyna.machut@gumed.edu.pl (K.M.); 2 Department of Orthodontics, Medical University of Lodz, 90-419 Lodz, Poland; elzbieta.pawlowska@umed.lodz.pl; 3 ORTODENT, Specialist Orthodontic Private Practice in Grudziadz, 86-300 Grudziadz, Poland

**Keywords:** plasma rich in growth factors, platelet rich fibrin, advanced platelet rich fibrin, apical periodontitis, endodontics, endodontic treatment, bone regeneration

## Abstract

Platelet concentrates have been widely used in regenerative medicine, including endodontics. The aim of this manuscript was to assess critically the efficacy of PRF in the treatment of endodontic periapical lesions in adult patients on the basis of the literature. The PICO approach was used to properly develop literature search strategies. The PubMed database was analyzed with the keywords: “((PRP) OR (PRF) OR (PRGF) OR (CGF)) AND (endodontic) AND ((treatment) OR (therapy))”. After screening of 155 results, 14 articles were included in this review. Different types of platelet concentrates are able to stimulate the processes of proliferation and differentiation of mesenchymal stem cells. Platelet rich fibrin (PRF) releases growth factors for at least 7 days at the application site. Growth factors and released cytokines stimulate the activity of osteoblasts. Moreover, the release of growth factors accelerates tissue regeneration by increasing the migration of fibroblasts. It was not possible to assess the efficacy of PRF supplementation in the treatment of endodontic periapical lesions in permanent, mature teeth with closed apexes, due to the lack of well-designed scientific research. Further studies are needed to analyze the effect of PRF on the healing processes in the periapical region.

## 1. Introduction

The term “periapical periodontium” encompasses all of the periradicular tissues localized in the area of the apex of the root, including periodontal ligaments, cementum and alveolar bone. Periapical periodontium is a part of the tooth suspension apparatus [1]. Apical periodontitis is the inflammatory process localized within the periapical periodontium, which is known as the host defense to the invasive microflora localized in the root canal system [2,3]. Nair describes the apical periodontitis as a dynamic encounter between root canal infection and host response [3]. There have been listed several different etiological factors which may cause the periapical periodontitis, namely: pulp pathology (infection, necrosis), tooth trauma, and also chemical, mechanical and bacterial factors related to the performed endodontic treatment (also known as root canal treatment) [2,3,4,5,6,7,8].

The onset of the inflammation within the periapical tissues depends on the time, the type of the pathological stimulus, and the susceptibility of the tissues [9]. Inflamed periapical tissues are considered to be free from bacteria [7]. However, there have also been published some studies, which confirm the presence of the bacteria biofilms on the root surfaces outside of the apical foramen. Noguchi et al. [10] detected Fusobacterium nucleatum (in all samples), Porphyromonas gingivalis (in 12 of 14 cases) and Tannellera forsythensis (in 8 of 14 samples) within the periradicular biofilms taken from the patients diagnosed with the resistant periapical periodontitis.

There are a few different treatment methods of chronic periradicular inflammatory processes, including root canal treatment (RCT), combination of conservative and surgical treatment (endodontic microsurgery), and sometimes, in non-prognostic cases—tooth extraction [4,8,11,12,13,14]. Moreover, some authors also recommend combination of abovementioned methods of treatment with the physical therapy (biostimulation with laser therapy, iontophoresis, induction of low magnetic field, i.e., magnetostimulation). In authors’ opinions additional physical therapy improves the effectiveness of a basic treatment and intensifies the regeneration processes and repairs [15,16,17,18].

Autologous platelets concentrate (APC) can be additionally used in the endodontic and surgical treatment, as well as after tooth extraction. APC has been found to be a biomaterial with a proven regeneration and repair improvement effect [19,20]. Platelets affect the process of angiogenesis by releasing growth factors from alpha-granules. The exemplary growth factors released form platelets alpha-granules are platelet-derived epidermal growth factor (PD-EGF), platelet-derived growth factor (PDGF), vascular endothelial growth factor (VEGF), basic fibroblast growth factor (bFGF) and transforming growth factor-beta (TGF-β) [21,22,23].

There can be obtained various types of APC, depending on the exact method of centrifugation. Platelet rich plasma (PRP) is an exemplary type of APC, which is formed by mixing platelet concentrate, derived from the centrifugation of autologous whole blood, with thrombin and calcium chloride [24]. Contrary to PRP, to obtain platelet rich fibrin (PRF) no additional anticoagulants are needed. Apart from platelets, PRF contains many blood cells, including B and T lymphocytes, monocytes, neutrophils, stem cells, as well as growth factors [25].

So far, PRF has been widely discussed in the field of oral surgery and pediatric dentistry. However, very little is known about the efficacy of PRF in the treatment of periapical lesions in mature, permanent teeth with closed apexes. Therefore, the aim of this manuscript was to assess critically the efficacy of PRF in the treatment of endodontic periapical lesions in adult patients on the basis of the literature.

## 2. Molecular Background of the Endodontic Periapical Lesions

### 2.1. Pro-Inflammatory and Anti-Inflammatory Cytokines

The majority of the studies acknowledge that the periapical inflammation with the following bone destruction are mainly induced by the cytokines secreted in response to the bacterial infection within the root canal. Bacteria stimulate the secretion of proinflamatory cytokines, inlcuding: IL-1β, IL-6, TNF-α [26,27,28].

There are pro-inflammatory and anti-inflammatory cytokines prsent within the periapical periodontitis. Both groups of cytokines play a significant role in the pathophisiology of the chronic inflammation in the area of periapical periodontium [29,30]. The pro-inflammatory cytokines (i.e., IL-1α, IL-2 or TNF- α) are mainly produced by the Th1 cells, macrophages and neutrophils. They take part in bacterially-induced bone resorption. In contrast, the anti-inflammatory cytokines (i.e., IL-4, IL-10) are produced by the Th2 cells and protect bone against destruction. Moreover, the anti-inflammatory cytokines participate in the healing process of the periapical lesions [28,29,30,31].

De Carvalho Fraga et al. [32] analyzed the levels of different cytokines in human radicular cysts and periapical granulomas. The authors found that IFN-γ concentrations were increased in radicular cysts, whereas the expression of IL-4 was stronger in periapical granulomas. Furthermore, Walker KF et al. [33] showed, that Th2 cells and their products predominated in periapical lesions. In authors’ opinion, the suppression of the inflammatory response combined with the increased number of Th2 lymphocyte subsets may directly affect the final outcome of the periapical pathological process.

Table 1 presents the general characteristics and functions of pro-inflammatory and anti-inflammatory cytokines which occur within the periapical lesions on the basis of the literature [28,29,34,35,36,37,38,39,40,41,42,43,44,45,46,47,48,49,50].

### 2.2. The Role of MicroRNA (miRNA) in Bone Resorption and Pathogenesis of Apical Periodontitis

MicroRNA (miRNA) is an endogenous, single-stranded, short (21 to 25 nucleotides), non-coding RNA that modulates post-transcriptional gene expression [51]. miRNA can bind to multiple mRNAs and lead to inhibition of protein translation or interfere with mRNA splicing. One mRNA can have multiple miRNA binding sites [52]. miRNA binds to the mRNA target sites, which are located in the 3′ untranslated regions (3′UTR). This binding may stop the expression of transcripts, and consequently reduce the proteins quantity [53,54].

Genes of miRNA are transcribed by RNA polymerase II into primary miRNAs (pri-miRNAs) [55]. pri-miRNAs are processed by Drosha/DGCR8 nuclear RNase III to a pre-miRNA. Then, this product is exported by Exportin-5 to the cytoplasm in a Ran-GTP-dependent manner. In the cytoplasm the pre-miRNAs are transformed by RNase III Dicer to acquire the mature miRNAs [56,57].

Here, miRNA may quickly reduce the amount of proteins present within the cell by lowering their expression from the transcripts. miRNAs regulate the majority of cellular processes, including cell division, differentiation, aging, the course of their metabolism and finally apoptosis [58,59]. Furthermore, there may be a link between the miRNA activity and regulation of inflammation and oxidative stress [60].

In this case, miRNA was also found to play a significant role in the pathogenesis of apical periodontitis. Chan et al. [61] compared the expression of miRNAs in periapical lesions and healthy control tissues (normal periodontal ligament and pulp tissues). The material from periapical lesions was curetted from the bony cavity during the procedure of apicoectomy, performed in teeth with non-healing periapical lesions after root canal treatment. The authors identified 381 different miRNAs in periapical tissues, among which 24 miRNAs were down-regulated. One of the very important miRNAs’ families, which appeared to be down-regulated, was the family of miR-181. The increase in the target messenger RNAs is the consequence of miRNAs downregulation. There have been listed several different miRNA targets, which affect both the inflammatory and immune response, including toll-like receptor-4 (TLR-4), interleukin-6 and -10 (IL-6, IL-10), chemokine ligand 8 (CCL8), transforming growth factor beta 1 (TGF-β1), vascular endothelial growth factor α (VEGF-α) and metalloproteinase-9 (MMP-9) [61]. The miR-181a, miR-181b and miR-181c, miR-24-1, miR-95, miR-149 and miR-455-3p were detected in altered periapical tissues [61].

miRNA is also believed to regulate osteoclastogenesis [62]. The mineralized bone matrix is degraded by osteoclasts. Metalloproteinases (MMPs), which are one of the targets of the miRNA, were found to be able to degrade bone organic matrix [61,63]. Lack of miRNA activity inhibits osteoclast precursor cells to produce osteoclasts. Without the presence of miRNAs, the number of osteoclasts becomes reduced. Therefore, the amount of resorbed bone is limited [62]. miRNA-21 is a well-known miRNA, that promotes osteoclastogenesis and osteogenic differentiation of mesenchymal stem cells [64,65]. It also regulates skin wound healing and affects collagen deposition [66]. Moreover, mi-RNA-21 is also a marker of chronic inflammation in diabetes mellitus (DM) [67]. miRNAs have been found to regulate the inflammation and oxidative stress, two processes which affect bone healing in the course of DM [65]. Baćević et al. [65] found that the usage of leukocyte- and platelet-rich fibrin (L-PRF) improved cranial bone healing in diabetics rabbits, by increasing expression levels of miRNA-21 and by reducing oxidative stress. It seems that L-PRF used either alone or with Bio-Oss as bone graft may be efficient in DM, because of affecting the expression of miRNA-21 [65].

Table 2 presents the exemplary miRNA occurring within the periapical lesions on the basis of the literature [61,68,69,70,71,72].

## 3. Endodontic Treatment of Periapical Lesions

Endodontic periapical lesions are typically one of the indications for an endodontic treatment. In case of larger lesions, with a diameter greater than or equal to 8 mm, it is recommended to perform two-visit therapy [8]. In two-stands treatment, the root canal is temporarily filled between visits, usually with a medication based on a non-hardening calcium hydroxide (Ca(OH)_2_).

Non-hardening Ca(OH)_2_ has a bactericidal effect, stimulates tissue mineralization and finally induces the processes of regeneration and repair of periapical lesions [73,74,75]. A temporary Ca(OH)_2_ filling is typically left in the root canal for 3 weeks, and should not be left for longer than 3 months. This period of time seems to be sufficient to observe healing of periapical lesions, both clinically and radiologically. Finally, after this period of time, the temporary filling material is removed from the root canal, and the endodontic treatment can be completed [8]. According to the recent researches, a long-lasting effect of Ca(OH)_2_ for many months is denied. To eliminate sufficiently microbiota from root canal, it is enough to temporarily fill the root canal with non-hardening Ca(OH)_2_ for one week [76]. Peters and Wesselink [77] compared the efficacy of two visit endodontic treatment (with the use of Ca(OH)_2_ as a temporal filler of the root canal) with the single visit root canal treatment (canals were obturated with gutta-percha and AH-26 sealer without the use of any temporal fillers). The authors did not notice any significant differences regarding the process of periapical lesions’ healing.

However, it should be emphasized that mechanical removal of infected dentin and proper irrigation with antibacterial agents (most commonly used: sodium hypochlorite (NaOCl), chlorhexidine (CHX), ethylenediaminetetraacetic acid (EDTA) and mixture of tetracycline, acid and detergent (MTDA)) lead to almost complete elimination of bacteria from the root canal system [78]. Rodrigues et al. [79] conducted the clinical trial among patients who underwent endodontic treatment of the teeth due to chronic periodontitis and found that chemomechanical root canal preparation is highly efficient method of root canal eradication (eradication at the level of 83.7 to 96.7%). The efficiency of the irrigation process may be even increased with the ultrasonic activation of irrigants [80]. The recommended protocol for the final root canal irrigation is the combination of 5.25% sodium hypochlorite (in two cycles with ultrasonic activation), 40% citric acid and distilled water to reduce sodium hypochlorite and to neutralize citric acid [81].

Orstavik [82] found that almost 85% of periapical lesions healed within 48 months. However, only 42.5% of the lesions were in the process of healing 6 months after the end of endodontic treatment. According to the European Society of Endodontology guidelines, it is recommended to observe the periapical lesion for at least 4 years before further, more radical procedures will be performed. Healing of the periapical lesions is considered to be a prolonged process [26].

When RCT is not successful, the surgical procedures are performed. The aim of the endodontic surgical procedure is to remove the root apex with the adjacent inflammatory periapical lesion. There are different surgical procedures used for the treatment of periapical lesions, including the resection of the root apex, and hemisection, which aims to remove one root (in case of multi-rooted teeth), leaving the remaining ones undamaged [83,84]. Unfortunately, there are several complications related to the endodontic surgical procedures, including excessive bone loss, bleeding, damage to the adjacent anatomical structures (e.g., nerve bundles), periodontal complications such as a gingival recession or formation of an astringent scar [85]. As an alternative to a conventional surgery, microsurgical procedures can be carried out with the use of operating microscopy. The biggest advantage of microsurgical endodontics is minimizing the amount of a removed bone tissue. Due to the careful revision of the operation area, it is possible to perform the procedure precisely, and reduce the incidence of complications [85,86,87]. In addition, the retro preparation field and selection of the ultrasonic tip allow a more conservative approach to preserve more bone tissues [88].

When none of the abovementioned groups of methods of treatment is successful, the tooth is qualified for extraction [89,90]. The missing tooth always needs to be restored to maintain the stability of the entire stomatognathic system. If the missing tooth is not restored, the neighboring and opposite teeth start moving towards the space after the extracted tooth. Therefore, the disturbances in occlusion become created [89,90,91].

## 4. Platelet Concentrates–General Characteristics, and Role in the Endodontic Treatment

Platelets (PLT, thrombocytes) are the smallest blood components with a diameter of about 2.5 μm. They are fragments of megakaryocyte cytoplasm, surrounded by a cell membrane, devoid of a cell nucleus. The remaining cell organelles are typical for eukaryotic cells. Thrombocytes are characterized by an active metabolism, a highly organized cytoskeleton, as well as a presence of specific intracellular granules and adhesive proteins located on the surface of the PLT. Thrombocytes are terminally differentiated cells [21,92].

Platelets play a significant role in hemostasis. After a blood vessel interruption, platelets participate in the processes of activation, adhesion and aggregation. They also release substances contained in their granules. Platelets are also effector cells of the innate immune system. They can bind pathogens directly or indirectly through the receptor proteins (including TLR2, TLR4, TLR7, TLR9, etc.), to present them to neutrophils and cells of the reticuloendothelial system [21,92,93]. The potential regenerative effect of platelets was first described by Ross et al. in 1974 [94].

In order to separate platelets from other blood elements, a centrifugation is carried out. Heavier elements fall down to the bottom of the tube, whereas the lighter ones float above. The lower layer consists of erythrocytes, while the upper one is composed of plasma. A platelet-leukocyte layer forms between these two fractions. The concentration of platelets in plasma is several times higher than in a non-centrifuged blood. This directly leads to an increased concentration of: PDGF, TGFβ, integrins and other adhesion molecules [23].

There are two major types of PRF according to the current classification of platelet concentrates from 2009: P-PRF (pure platelet rich fibrin) and L-PRF (leukocyte and platelet rich fibrin) [95].

P-PRF, also known as leukocyte-poor platelet-rich fibrin, is an autologous material without leukocytes and with a high-density fibrin network. It only exists in a strongly activated gel form, therefore it cannot be injected. On the basis of the literature, there is currently only one commercially available P-PRF product—Fibrinet PRFM (Platelet-Rich Fibrin Matrix, Cascade Medical, Wayne, NJ, USA), which is mostly used in orthopedics and sports medicine. The huge disadvantage of using P-PRF is its cost comparing to L-PRF [95,96,97].

Leukocyte and platelet rich fibrin (L-PRF) consists of leukocytes and a high-density fibrin network. There are two subcategories of L-PRF: A-PRF (advanced platelet rich fibrin) and i-PRF (injectable platelet rich fibrin) [96,97]. Their newest generations are named: A-PRF+ and i-PRF+. The novelty in obtaining platelet-rich fibrin refers to reduced centrifugation speed and time [98,99].

Newly invented PRF centrifugation protocols improve tissue regeneration [100]. The standard PRF is centrifuged at 2700 rpm for 12 min. Whereas to obtain the A-PRF, the blood is centrifuged at lower speeds, namely: 1500 rpm for 14 min. This modification of the centrifugation protocol leads to significant increase in the release of growth factors (PDGF, TGF-β1, epidermal growth factor (EGF), insulin-like growth factor (IGF)) in the application area, with a higher number of progenitor cells [99,100,101].

The main role of fibrin in the process of healing is hemostasis. However, it also regulates migration of fibroblasts and endothelial cells, which are involved in angiogenesis and are responsible for the new tissue formation. An important feature of PRF is the prolonged release of growth factors for at least 7 days at the application site [98]. Growth factors and released cytokines stimulate the activity of osteoblasts. The release of growth factors accelerates tissue regeneration by increasing the migration of fibroblasts [92,98,102,103]. VEGF stimulates angiogenesis, which is a crucial part of bone regeneration, as the blood supply promotes osteogenesis. It has been shown that angiogenesis occurs before osteogenesis in the healing of bone defects. VEGF can induce the mobilization, recruitment, proliferation and differentiation of endothelial progenitor cells (EPCs) as well osteoblast recruitment and survival [104].

The leukocytes within L-PRF present antimicrobial properties. They can act directly (i.e., phagocytosis) [105] or indirectly, presenting immunomodulatory activity (i.e., antibodies production) [106]. Kour et al. [107] found that three different platelet concentrates, namely: PRP, PRF and i-PRF, show antibacterial ability against periopathogens present in the oral cavity, including Porphyromonas gingivalis (P.g.) and Aggregatibacter actinomycetemcomitans (A.a.). The authors measured the width of inhibition zones to assess the antimicrobial properties of particular platelet concentrates. In case of P.g., i-PRF and PRP had significantly wider zones of inhibition comparing to PRF. In case of A.a., PRP had bigger zone of inhibition than PRF and i-PRF. Regarding the antibacterial properties against A.a., there were no significant differences between PRF and i-PRF [107].

Platelet concentrates, including PRF and PRP, are widely used in regenerative medicine, as well as in dentistry [108,109]. PRF can be used alone or in combination with other biomaterials. There are several different uses of platelet concentrates in dentistry, including: endodontic treatment of permanent teeth with incomplete apex development (pulpotomy, revascularization and apexification), guided bone regeneration (alveolar bone augmentation, sinus lifting), periodontal treatment (plastic periodontology surgery, treatment of bone loss defects). Moreover, the use of PRF in guided tissue regeneration scaffold has been the subject of extended investigations [110,111,112,113,114,115].

## 5. Mesenchymal Stem Cells and Platelet Concentrates

Mesenchymal stem cells (MSCs) are pluripotent, which means they can differentiate into various types of cells, i.e., osteoblasts, chondrocytes or adipocytes [116]. MSCs suppress the immune response. MSCs reduce the lymphocytes’ immunological responses, affect antigen-presenting cells by inhibiting their maturation, and finally reduce the immune activity of NK cells [116,117]. The above presented features of MSCs allow mesenchymal cells to take part in the regeneration processes [116,118]. There are several different types of stem cells involved into the regeneration processes of dental pulp cells, including stem cells of the apical papilla (SCAPs), periodontal ligament stem cells (PDLSCs) and human dental pulp cells (hDPCs) [119,120]. It has already been proven that autologous platelet concentrate may be a carrier for bone marrow mesenchymal stem cells (BMSCs) [121] and may stimulate the processes of proliferation and differentiation of BMSCs [122]. Sequeira et al. [123] used PRP as a scaffold for SCAPs and found that SCAPs induced formation of dentin-like and pulp-like tissues.

Xu et al. [124] analyzed the effects of concentrated growth factor (CGF) on human dental pulp stem cells (hDPSCs), which were exposed to lipopolysaccharide (LPS). The authors found that LPS stimulates the expression of proinflammatory cytokines in hDPSCs. Dental pulp cells chronically exposed to proinflammatory cytokines lose their ability to differentiate to osteoblasts. Xu et al. [124] noticed that CGF limited the release of proinflammatory cytokines and stimulated proliferation, migration and differentiation of hDSPCs. Rochira et al. [125] observed that CGF alone can stimulate osteogenic differentiation of human Bone Marrow Stem Cells (hBMSCs). According to the study by Zhang et al. [126], both the CGF and L-PRF release growth factors, including bFGF, TGF-β1 and bone morphogenetic protein 2 (BMP-2), which affect the differentiation of mesenchymal stem cells and lead to bone formation. The authors noticed that BMP-2 is released slowly for at least 7 days in CGF and L-PRF. Finally, Li et al. [127] summarized that CGF stimulates proliferation of different types of mesenchymal stem cells, including DPSCs, PDLSCs, as well as human mesenchymal stem cells (hTERT-E6/E7) in a dose-dependent manner.

## 6. PRF in the Endodontic Treatment of Permanent Teeth with Closed Apexes

The clinical effects of conventional RCT with an additional supplementation of PRF in the treatment of permanent teeth with closed apexes, diagnosed with periapical lesions, have been examined only by Machut et al. [128]. The authors presented two case reports with satisfactory bone healing 6 months after the end of RCT. The authors applied A-PRF membrane by the apical foramen to the periapical area. Permanent, mature teeth with closed apexes have mostly been treated with different types of PRF used as bone fillers (either alone or mixed with bone substitutes), and/or membranes placed over the bone defects in terms of endodontic surgery.

Soto-Peñaloza et al. [129] presented randomized clinical trial, which assessed the clinical effects of the use of A-PRF+ membranes in the endodontic surgery. This is the only one randomized clinical trial which analyzes the outcomes of PRF use in endodontic treatment of permanent teeth with closed apexes. The authors did not observe significant differences between the groups regarding postoperative pain. Moreover, only sleep and speech functions presented more limitations in control group.

All of the published case reports [130,131,132,133,134,135,136,137,138,139,140,141] revealed satisfactory bone healing after placing PRF or PRF mixed with bone substitutes inside the bone defects. However, they do not explain and answer the fundamental question if additional use of PRF in endodontic surgery is more beneficial for the patient than the conventional surgical procedures performed without any additional supplementation. Only Parikh et al. [141] compared the results of RCT and curettage of the defect with the results of RCT and curettage of the defect and additional PRF gel supplementation, performed in two upper central incisors in one patient. The authors noticed that additional use of PRF led to better bone healing.

Table 3 presents the use of PRF in the endodontic treatment of permanent teeth with closed apexes on the basis of the literature [128,129,130,131,132,133,134,135,136,137,138,139,140,141].

## 7. Materials and Methods

### 7.1. Clinical Question

What is the clinical efficacy of plasma rich in growth factors (PRGF) applied in the periapical region supplementary to conventional root canal treatment (RCT) of permanent teeth with closed apexes, diagnosed with periapical lesions, on the basis of the literature?

### 7.2. Inclusion and Exclusion Criteria for the Narrative Review

Table 4 presents inclusion and exclusion criteria for the narrative review.

### 7.3. The PICO Approach

We used the PICO approach to properly develop literature search strategies for this review:

Population: adolescents and adult patients; only mature teeth with closed apexes diagnosed with periapical lesion.

Intervention: RCT with additional application of PRGF or RCT with endodontic surgery with additional direct application of PRGF.

Comparison: RCT without additional application of PRGF; RCT with endodontic surgery without additional application of PRGF; placebo.

Outcome: healing of the periapical lesion and tooth pain reduction (in case of symptomatic periapical periodontitis).

### 7.4. Search Strategy

The PubMed database was analyzed with the following keywords: ((PRP) OR (PRF) OR (PRGF) OR (CGF)) AND (endodontic) AND ((treatment) OR (therapy)). After screening of 155 results, 14 studies were included in this review (one randomized controlled trial and 13 case reports).

**Figure 1:**
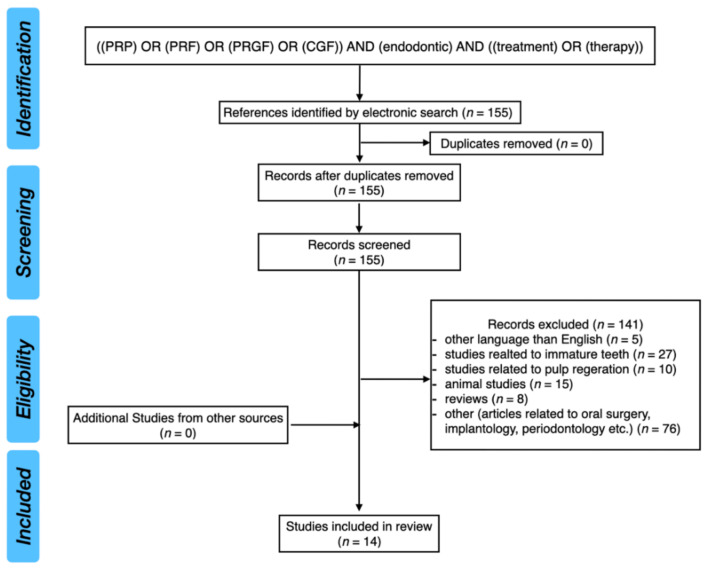
PRISMA flow diagram for review of the literature.

Figure 1 presents the PRISMA flow diagram for a review of the literature.

## 8. Conclusions

Different types of platelet concentrates, especially L-PRF and CGF, are able to stimulate the processes of proliferation and differentiation of mesenchymal stem cells. They induce the bone regeneration processes. Moreover, it has also been proven that L-PRF may affect the expression of miRNA-21.

Despite the fact, that PRF seems to promote bone healing and to reduce healing time, it was not possible to assess the efficacy of PRF supplementation in the treatment of endodontic periapical lesions in permanent, mature teeth with closed apexes, due to the lack of well-designed scientific research. Further studies, especially randomized double-blind controlled trials, are needed to assess whether the RCT with additional application of PRF is superior to RCT performed alone in the treatment of permanent mature teeth with closed apexes diagnosed with periapical lesions.

## Figures and Tables

**Table 1:** General characteristics and functions of pro-inflammatory and anti-inflammatory cytokines which occur within the periapical lesions on the basis of the literature [28,29,34,35,36,37,38,39,40,41,42,43,44,45,46,47,48,49,50].

Cytokine (Abbreviation)	Cytokine Receptors	Cytokine-Secreting Cells	Target Cells	Functions	Bone Effect
Interleukin-1 alpha (IL-1α)	Interleukin-1 receptor (IL-1R): type I (IL1R1) and type II (ILL1R2)	monocytes, macrophages, polymorphonuclear leucocytes (PMNs), fibroblasts, osteoclasts, epithelial cells, endothelial cells, B cells	T-cells, B-cells, neutrophils, osteoblasts, tissue cells	Induces the inflammation and regulates immune system by chemotactically activation of PMN. Stimulates the production of PG, proteolytic enzymes and proinflammatory cytokines IL-6, IL-8.	Bone destruction: stimulates bone resorption and inhibits bone formation. Inhibits osteoblasts differentiation and probably induces apoptosis of osteoblasts.
Interleukin-1 beta (IL-1β)	Interleukin-1 receptor (IL-1R): type I (IL1R1) and type II (ILL1R2)	macrophages, dendritic cells, osteoblasts, fibroblasts (i.e., gingival fibroblasts, periodontal ligament cells), osteoblasts, epithelial and endothelial cells	T-cells, fibroblasts, epithelial cells, endothelial cells	Induces the inflammation: accelerates blood flow in inflamed tissue, supports leucocyte recruitment and neutrophil diffusion and accumulation.	Bone destruction: promotes bone resorption by stimulating production of MMPs, (mainly MMP-9), RANKL, IL-6.
Interleukin-18 (IL-18)	Interleukin-18 receptor (IL-18R, CD218a)	macrophages, dendritic cells, monocytes, keratinocytes, CNS cells, osteoblasts, endothelial cells	T-cells (CD4 and CD8), NK-cells, basophils, mast cells	Induces the production of IFNγ by T-cells and NK-cells. Induces Th cell-mediated immunity. Promotes proliferation of Th1.	Bone destruction: promotes osteoclastogenesis by regulation of RANKL production.
Interleukin-6 (IL-6)	Interleukin-6 receptor (IL-6R, CD126)	monocytes, polymorphonuclear leucocytes (PMNs), osteoclasts, macrophages, T-cells (Th2), B-cells, fibroblasts, endothelial cells	T-cells, B-cells, neutrophils, osteoblasts, tissue cells	Acute phase of inflammation: activates PMNs and T-cells. Stimulates B-lymphocytes differentiation into plasma cell. Induces protein synthesis. Suppresses the production of IL-1.	Bone destruction: induces bone resorption by promoting osteoclast differentiation.
Interleukin-8 (IL-8)	Interleukin-8 receptor A (IL-8RA, CXCR1) and interleukin-8 receptor B (IL-8RB, CXCR2)	monocytes, macrophages, PMNs, bone marrow stromal cells, osteoblasts, osteoclasts, synovial fibroblasts, chondrocytes	neutrophils, basophils	Chemotactic factor: attracts and activates PMNs and osteoclasts.	Bone destruction (potentially): stimulates osteoclastogenesis by osteoclasts differentiation and production, by stimulating RANKL expression and directly by stimulation of osteoclasts pro-duction and activation.
Interleukin-10 (IL-10)	Interleukin-10 receptor: (IL-10R) type I (IL-10R1) and type II (IL-10R2)	T-cells, monocytes, dendritic cells, B-cells, mast-cells, eosinophils	Th1, macrophages, NK-cells	Inhibits the production of cytokines by Th1: IL-1, IL-6 and IFNγ. Inhibits synthesis of NO and proteases (such as collagenases). Stimulates the secretion of tissue inhibitors of metalloproteinases and osteoprotegerin.	Inhibits bone resorption, suppresses the osteoclastogenesis and activates proliferation of osteoblasts.
Interleukin-17	Interleukin-17 receptor (IL-17R)	Th17, Tregs	T-cells, B-cells, osteoblasts, tissue cells	Induces the inflammation. Activates secretion of IL-1, IL-6, TNFα, GCP-2 and IL-8. Induces migration of neutrophils.	Bone destruction: stimulates bone resorption, stimulates the production of RANKL by osteoblast and mesenchymal stem cells, disturbs balance of RANKL/OPG, which promotes osteoclastogenesis.
Tumor Necrosis Factor α (TNFα)	Tumor necrosis factor receptor 1 (TNFR1, CD120a); Tumor necrosis factor receptor 2 (TNFR2, CD120b)	macrophages, monocytes, lymphocytes (Th1), mast cells	macrophages, granulocytes, endothelial cells	Induces the inflammation by activating lymphocytes and monocytes.	Bone destruction: stimulates bone resorption, supports osteoclastogenesis with RANKL, promotes differentiation of osteoclasts and suppresses formation of osteoblasts.
Interferon gamma (IFNγ)	Interferon gamma receptor 1 (IFNGR1, CD119) and Interferon gamma receptor 2 (IFNGR2)	T-cells (CD4+, CD8+), Treg cells, B-cells, NK cells	monocytes, lymphocytes, tissue cells, mesenchymal stem cells (MSCs)	Activation of macrophages and differentiation of B-cells. Induces production of IL-1, NO and oxygen.	Inhibits bone resorption: inhibits production and differentiation of osteoclasts, activates apoptosis of osteoclasts. Indirectly down-regulates RANKL-depended osteoclastogenesis. Promotes differentiation of osteoblast from MSCs.
Interleukin-4 (IL-4)	Interleukin-4 receptor (IL-4, CD124)	Th2	Th17	Suppresses Th17 formation and production of IL-1. Stimulates the secretion of tissue inhibitors of metalloproteinases and osteoprotegerin.	Inhibits bone resorption, inhibits the osteoclast differentiation. It may promote osteoprotegerin pro-duction.
Granulocyte-Macrophage Colony Stimulating Factor (GM-CSF aka CSF2)	GM-CSF receptor (GM-CSFR)	macrophages, mast cells, T-cells, fibroblasts, NK cells, endothelial cells	bone marrow stem cells, macrophages, neutrophils	Takes part in hematopoiesis. Induces production of granulocytes (neutrophils, basophils, eosinophils) and monocytes from bone marrow stem cells. Activates macrophages. Enhances neutrophils migration.	Inhibits formation of osteoclasts from progenitor cells, reduces the RANKL/RANK activity. The increased level of dendritic cells makes GM-CSF activate osteoclastogenesis.

B-cell–lymphocyte type B, CD–cluster of differentiation, CNS cells–central nervous system cells, GCP-2–granulocyte chemotactic protein–2, IFNγ–interferon γ, IL–interleukin, MMP–matrix metalloproteinase, MSC–mesenchymal stem cell, NK–natural killer T-cell, NO–nitrous oxide, OPG–osteoprotegerin, PG–prostaglandin, PMN–polymorphonuclear leucocytes, RANK–Receptor Activator for Nuclear Factor κ B, RANKL–Receptor Activator for Nuclear Factor κ B Ligand, T-cell–lymphocyte type T, Th–T helper lymphocyte, TNFα–tumor necrosis factor α, Treg cell–regulatory T cell.

**Table 2:** Exemplary miRNA occurring within the periapical lesions on the basis of the literature [61,68,69,70,71,72].

MicroRNA (miRNA)	Gene	Effect
miRNA-155	21q21.3	Inhibition of SEMA3A. Decreased expression of SEMA3A contributes to bone resorption.
miRNA-335-5p	7q32.2	In inflamed HPDLFs promotes bone resorption (RANKL).
miRNA-181b-5p	1q32.1	Positive regulation of: acute inflammation (activation of NK-cells, monocytes, T-cells), angiogenesis, macrophages differentiation. Cementoblasts apoptosis.
miRNA-146a	5q33.3	Anti-inflammatory activity. Negative regulation of IL-6, IL-1β and TNF-α.
miRNA-10a-5p	17q21.32	Reduction of inflammation. Healing of apical periodontitis.

SEMA3A–Semaphorin 3A, HPDLFs–Human Periodontal Ligament Fibroblasts, RANKL–Receptor Activator for Nuclear Factor κ B Ligand, T-cell–lymphocyte type T, NK–natural killer T-cell, IL-6–Interleukin 6, IL-1β–Interleukin 1 beta, TNFα–Tumor Necro-sis Factor.

**Table 3:** PRF in the endodontic treatment of permanent teeth with closed apexes on the basis of the literature [128,129,130,131,132,133,134,135,136,137,138,139,140,141].

Reference	Study Design	Participants and Intervention	Endpoint and Results
Machut et al. (2021) [128]	Case report	*Case no 1:* Patient: 45-year-old female Tooth/teeth: 23 Diagnosis: pulp necrosis with symptomatic apical periodontitis of tooth 23 Type of treatment: RCT with A-PRF membrane placed by the apical foramen to the periapical area Protocol to obtain A-PRF: (1) 10 mL of venous blood was drawn (2) centrifugation: 1200 rpm for 8 min *Case no 2:* Patient: 42-year-old male Tooth/teeth: 23 Diagnosis: pulp necrosis with asymptomatic apical periodontitis of tooth 23 Type of treatment: RCT with A-PRF membrane placed by the apical foramen to the periapical area Protocol to obtain PRF: (1) 10 mL of venous blood was drawn (2) centrifugation: 1200 rpm for 8 min	*Endpoint:* 6 months Additional application of A-PRF led to a significant decrease in the periapical lesions’ size.
Soto-Peñaloza et al. (2020) [129]	Randomized clinical trial	Patient: 50 patients who needed endodontic surgery of upper maxillary teeth; age range: 44.2–52.4 years old Tooth/teeth: upper maxillary teeth (second premolar to second premolar) Diagnosis: chronic apical periodontitis Type of treatment: RCT + endodontic surgery (resection) Retrograde filling material: MTA Bony defect filler: control group: n/a; study group: A-PRF+ membranes (approximately: 2 + additional 1 over the osteotomy) Protocol to obtain PRF: (1) venous blood was drawn (no information about amount) (2) centrifugation: 1300 rpm for 8 min (tubes without AC) Sutures: 4–0 polyamide	*Endpoint:* 7 days No significant differences between the groups regarding postoperative pain. Only sleep and speech functions presented more limitations in control group.
Kavitha et al. (2020) [130]	Case report	*Case no 1:* Patient: 23-year-old female Tooth/teeth: 11,12 Diagnosis: chronic periapical abscess in relation to nonvital teeth: 11, 12 Type of treatment: RCT + endodontic surgery (resection) Retrograde filling material: Glass Ionomer Cement (GC Fuji IX) Bony defect filler: PRP + small amount of bovine thrombin + a few drops of 10% calcium chloride + β-TCP Protocol to obtain PRP: (1) 10 mL of venous blood was drawn (2) centrifugation: 5000 rpm for 15 min (tubes with EDTA) (3) second centrifugation of superior plasma: 2000 rpm for 10 min (4) PPP was discarded Sutures: 3–0 nonabsorbable black silk *Case no 2:* Patient: 23-year-old female Tooth/teeth: 22 Diagnosis: periapical abscess in relation to nonvital 22 Type of treatment: RCT + endodontic surgery (resection) Retrograde filling material: Glass Ionomer Cement (GC Fuji IX) Bony defect filler: PRF + β-TCP Protocol to obtain PRF: (1) 10 mL of venous blood was drawn (2) centrifugation: 3000 rpm for 15 min (tubes without AC) Sutures: 3–0 nonabsorbable black silk	*Endpoint:* 1 year Both PRP and PRF mixed with β-TCP were effective in the treatment of periapical defects.
Sureshbabu et al. (2020) [131]	Case report	Patient: 26-year-old male Tooth/teeth: 43,44,45 Diagnosis: pulpal necrosis with a chronic apical abscess in 43, 44, 45 Type of treatment: RCT + endodontic surgery (resection) Retrograde filling material: MTA Bony defect filler: CGF + osseograft + CGF membrane Protocol to obtain CGF: (1) 20 mL of venous blood was drawn (2) acceleration for 30 s; centrifugation at 2700 rpm for 2 min, 2400 rpm for 4 min, 2700 rpm for 4 min, 3000 rpm for 3 min; deceleration for 36 s (3) centrifugation of superior plasma: 2000 rpm for 10 min Sutures: 3–0 vicryl	*Endpoint:* 1 year and 2 years After 1 year, lesion reduction size was found to be 79%.
Taschieri et al. (2012) [132]	Case report	Patient: 28-year-old male Tooth/teeth: 21,22 Diagnosis: periradicular lesion of endodontic origin, vestibular sinus tract and an abscess on the palatal side, nonvital tooth 22, tooth 21 after RCT with large endodontic post Type of treatment: RCT + endodontic surgery (resection) Retrograde filling material: n/a Bony defect filler: PRGF + 50 μL of 10% CaCl2 were added per cubic centimeter of PRGF concentrate + Bio-Oss mixed with PRGF + BioGide membrane Protocol to obtain PRGF: (1) 5 mL of venous blood was drawn (2) centrifugation: 460× *g* for 8 min (tubes with 3.8% trisodium citrate) Sutures: non-absorbable silk 5–0	*Endpoint:* 1 year After 1 year the authors noticed complete healing and functionality.
Shivashankar et al. (2013) [133]	Case report	Patient: 45-year-old male Tooth/teeth: 12,11 Diagnosis: exacerbated chronic periodontitis in relation to nonvital teeth: 11, 12 Type of treatment: RCT + endodontic surgery (resection) Retrograde filling material: MTA Bony defect filler: PRF + HA bone graft crystals + PRF membrane Protocol to obtain PRF: (1) 20 mL of venous blood was drawn (2) centrifugation: 3000 rpm for 10 min Sutures: 3–0 black silk suture material	*Endpoint:* 2 years The authors noticed complete bone healing after 2 years.
Zhao et al. (2014) [134]	Case report	*Case no 1:* Patient: 28-year-old female Tooth/teeth: 13,12,21,22 Diagnosis: exacerbated chronic periodontitis in relation to nonvital teeth: 13,12, 21,22; incomplete root canal fillings of teeth no: 12,21,22 Type of treatment: RCT + endodontic surgery (resection) Retrograde filling material: amalgam Bony defect filler: minced PRF mixed with resorbable bioactive glass + PRF membrane Protocol to obtain PRF: (1) venous blood was drawn (no information about amount) (2) centrifugation: 3000 rpm for 12 min (tubes without anticoagulant) Sutures: 4–0 silk *Case no 2:* Patient: 27-year-old female Tooth/teeth: 14,13,12,21,22 Diagnosis: exacerbated chronic periodontitis in relation to nonvital teeth: 14,13, 12,11,21; incomplete root canal fillings of teeth no: 14,13,12,11,21 Type of treatment: RCT + endodontic surgery (resection) Retrograde filling material: amalgam Bony defect filler: minced PRF mixed with resorbable BG + PRF membrane Protocol to obtain PRF: (1) venous blood was drawn (no information about amount) (2) centrifugation: 3000 rpm for 12 min (tubes without anticoagulant) Sutures: 4–0 silk	*Endpoint:* 7 months (1st case) 4 months (2nd case) The authors noticed satisfactory bone healing at the endpoint examination.
Dudeja et al. (2017) [135]	Case report	Patient: 26-year-old female Tooth/teeth: 21,22 Diagnosis: chronic periodontitis in relation to nonvital teeth: 21,22; suppurative sinus tract between teeth 22 and 23 Type of treatment: RCT + endodontic surgery (resection) Retrograde filling material: MTA Bony defect filler: PRF membrane + PRF mixed with an irradiated FDBA + collagen membrane Protocol to obtain PRF: (1) venous blood was drawn (no information about amount) (2) centrifugation: n/a Sutures: 3–0 silk	*Endpoint:* 1 year After 1 year the authors observed continuation of healing process and decrease in the size of radiolucency.
Wadhwa et al. (2017) [136]	Case report	Patient: 25-year-old male Tooth/teeth: 46 Diagnosis: exacerbated chronic periodontitis in relation to nonvital tooth: 46 Type of treatment: RCT + endodontic surgery (resection) Retrograde filling material: MTA Bony defect filler: PRF membrane Protocol to obtain PRF: (1) 10 mL of venous blood was drawn (2) centrifugation: 3000 rpm for 10 min (tube without anticoagulant) Sutures: 4–0 monofilament	*Endpoint:* 18 months After 18 months the authors observed successful outcome of the healing process.
Vidhale et al. (2015) [137]	Case report	Patient: 22-year-old male Tooth/teeth: 21,22,23 Diagnosis: exacerbated chronic periodontitis in relation to nonvital teeth: 21,22, 23, radicular cyst Type of treatment: RCT + endodontic surgery (resection) Retrograde filling material: n/a Bony defect filler: PRF + iliac bone graft Protocol to obtain PRF: (1) 5 mL of venous blood was drawn (2) centrifugation: 3000 rpm for 10 min (tube without anticoagulant) Sutures: n/a	*Endpoint:* 3 months The authors noticed the presence of bone healing.
Bains et al. (2012) [138]	Case report	Patient: 39-year-old male Tooth/teeth: 46 Diagnosis: retrograde periodontitis along with Grade II furcation involvement with definitive pulpal perforation in the tooth 46, incomplete root canal treatment of tooth 47 Type of treatment: RCT + regenerative periodontal surgery Retrograde filling material: n/a Bony defect filler: PRF gel mixed with hydroxyapatite graft material + PRF membrane Protocol to obtain PRF: (1) 10 mL of venous blood was drawn (2) centrifugation: 3000 rpm for 12 min (tube without anticoagulant) Sutures: n/a	*Endpoint:* 1.5 years The authors noticed significant limitation of the radiolucency area.
Demiralp et al. (2004) [139]	Case report	Patient: 45-year-old male Tooth/teeth: 11,21 Diagnosis: exacerbated chronic periodontitis in relation to nonvital teeth: 11,21 Type of treatment: RCT + surgery (scaling and root-planning, resection was not performed) Retrograde filling material: n/a Bony defect filler: PRP gel + PRP gel mixed with TCP + PRP gel Protocol to obtain PRP: (1) 30 mL of venous blood was drawn (2) centrifugation: n/a Sutures: 4–0 silk	*Endpoint:* 1 year The authors noticed almost complete resorption of TCP particles and new bone formation.
Hiremath et al. (2014) [140]	Case report	Patient: 20-year-old male Tooth/teeth: 11,21,22 Diagnosis: exacerbated chronic periodontitis in relation to nonvital teeth: 11,21,22 Type of treatment: RCT + surgery (curettage of the defect) Retrograde filling material: n/a Bony defect filler: PRF mixed with HA Protocol to obtain PRP: (1) venous blood was drawn (no information about amount of blood, tube without anticoagulant) (2) centrifugation: 571.54× *g* for 12 min Sutures: 4–0 silk	*Endpoint:* 18 months After 18 months the authors observed complete bone healing.
Parikh et al. (2011) [141]	Case report	Patient: 24-year-old male Tooth/teeth: 11,21 Diagnosis: exacerbated chronic periodontitis in relation to nonvital teeth: 11,21 Type of treatment: RCT + surgery (curettage of the defect) Retrograde filling material: MTA Bony defect filler: PRP gel was placed at the site of larger defect (left side) Protocol to obtain PRP: (1) 8.5 mL of venous blood was drawn (tube with anticoagulant) (2) centrifugation: n/a Sutures: 4–0 Mersilk	*Endpoint:* 2 years After 8 weeks the authors observed better bone healing at the site treated with PRP.

AC–anticoagulant, BG–bioactive glass, β-TCP–β-tricalcium phosphate, CGF–concentrated growth factor, EDTA–Ethylene diamine tetraacetic acid, FDBA–freeze-dried bone allograft, HA–hydroxyapatite, MTA–Mineral Trioxide Aggregate, n/a–not applicable, PPP–platet poor plasma, RCT–root canal treatment, TCP–tricalcium phosphate.

**Table 4:** Inclusion and exclusion criteria for the narrative review.

Criteria	List of Specific Criteria
Inclusion criteria	randomized controlled trials
	randomized clinical trials
	case reports
	case–control studies
	study population: adult patients (aged: 18 years old or more); permanent teeth with closed apexes, diagnosed with periapical lesions
	methods of treatment: RCT with application of PRGF
Exclusion criteria	systematic reviews and metanalyses
	comments
	animal studies
	study population: children with deciduous teeth, children and adolescents with immature teeth (teeth with opened apexes), teeth without periapical lesions
	methods of treatment: endodontic procedures without application of PRGF, endodontic surgery
	papers written in languages other than English

PRGF—plasma rich in growth factors; RCT–root canal treatment.

## Data Availability

All data are contained within the article.
